# The influence of 3D printing on inter- and intrarater reliability on the classification of tibial plateau fractures

**DOI:** 10.1007/s00068-022-02055-1

**Published:** 2022-08-09

**Authors:** Tobias Dust, Maximilian J. Hartel, Julian-Elias Henneberg, Alexander Korthaus, Tobias Malte Ballhause, Johannes Keller, Malte Ohlmeier, Kai-Jonathan Maas, Karl-Heinz Frosch, Matthias Krause

**Affiliations:** 1 Department of Trauma and Orthopaedic Surgery, University Medical Center Hamburg-Eppendorf, Martinistrasse 52, 20246 Hamburg, Germany; 2 Department of Trauma Surgery, Orthopaedics and Sports Traumatology, BG Hospital Hamburg, Hamburg, Germany; 3 Department of Diagnostic and Interventional Radiology and Nuclear Medicine, University Medical Center Hamburg-Eppendorf, Hamburg, Germany

**Keywords:** Tibial plateau fracture, 3D printing, Diagnostics, AO classification, 10-segment classification, Revisited Schatzker classification

## Abstract

**Purpose:**

Tibial plateau fractures continue to be a challenging task in clinical practice and current outcomes seem to provide the potential for further improvement. Especially presurgical understanding of the orientation of fracture lines and fracture severity is an essential key to sufficient surgical treatment. The object of this study was to evaluate the reliability of modern axial CT-based classification systems for tibial plateau fractures. In addition, the diagnostic-added value of 3D printing on the classification systems was investigated.

**Methods:**

22 raters were asked to classify 22 tibial plateau fractures (11 AO B- and 11 AO C-fractures) with the AO, the 10-Segment and the Revisited Schatzker classification in a three-step evaluation: first only using CT scans, second with 3D volumetric reconstructions and last with 3D-printed fracture models. Inter- and intraobserver agreement and the subjective certainty were analyzed. Statistics were done using kappa values, percentage match and a univariant one-way analysis of variance.

**Results:**

The AO classifications interobserver percentage match and kappa values improved for all raters and recorded an overall value of 0.34, respectively, 43% for the 3D print. The 10-Segment classification interobserver agreement also improved with the 3D-printed models and scored an overall kappa value of 0.18 and a percentage match of 79%. Equally the Revisited Schatzker classification increased its values to 0.31 and 35%. The intraobserver agreement showed a moderate agreement for the AO (0.44) and Revisited Schatzker classification (0.42) whereas the 10-Segment classification showed a fair agreement (0.27). Additionally, the raters changed their classification in 36% of the cases after evaluating the fracture with the 3D-printed models and the subjective certainty regarding the decisions improved as categories of self-reliant diagnostic choices were selected 18% (*p* < 0.05) more often after using the 3D-printed models.

**Conclusion:**

Based on the measured outcomes it was concluded that the new classification systems show an overall slight to fair reliability and the use of 3D printing proved to be beneficial for the preoperative diagnostics of tibial plateau fractures. The 10-Segment classification system showed the highest percentage match evaluation of all classification systems demonstrating its high clinical value across all levels of user experience.

## Introduction

The operative treatment of tibial plateau fractures remains to be a challenging task and current outcomes seem to provide the potential for further improvement [1–4]. For optimal final results, next to the injury mechanism and the underlying soft tissue trauma, a thorough study of the radiographic imaging material is paramount in the preparation of the surgical strategy [1, 5, 6]. As practiced in many other anatomic regions, classification systems provide one major foundation of surgical decision making in tibial plateau fractures [7]. Classification systems are each based on the technology at hand at the time of their development. Traditional classification systems (AO, Schatzker, Moore) that categorize different fracture patterns at the tibial plateau were based on two-dimensional plain radiographs [7, 8]. However, their poor reliability limits their benefit in clinical reality [9]. Over time, with newer technologies, such as computed tomography emerging, modern classification systems incorporated the third (axial) dimension to be able to process the additional information provided [10–12]. Hence classifications of fracture patterns have become more reliable but still lack consensus [13]. The concept of current CT-based classifications is not only the analysis of fracture patterns. They also help the user in the decision making on the selection of the correct surgical approaches and osteosynthetic materials [11, 14].

But, a comparison of modern axial fracture classifications in terms of reliability testing as primary endpoint has not been performed, yet. To date, it remains unclear whether new technologies such as 3D imaging, modeling and printing can provide additional contribution to diagnostic confirmation and surgical planning, especially in clinical application of modern CT-based classification systems [15, 16]. These may have the potential to further improve preoperative preparation routines and possibly classification accuracy, too [15, 17].

The hypothesis was that the choice of the particular CT-based classification system significantly influences the diagnosis and operative management strategy of tibial plateau fractures among surgeons with different levels of experience. In addition, the impact of 3D printing technology on the inter and intraobserver reliability of the modern classification systems was investigated.

## Methods

Twenty-two cases with an intra-articular tibial plateau fracture AO/OTA Type B or C and a computed tomography (CT) with an axial section slice thickness of 1 mm or less were included in this study. There were analyzed by 22 raters, with different levels of experience. There were 5 medical students, 10 resident doctors, 3 consultants with an average level of experience in tibial plateau fracture surgery (“junior surgeons”) and 4 senior surgeons with expert level experience.

### Image acquisition

Computed tomography scans of tibial plateau fractures were exported from the institution’s PACS System and saved in DICOM (Digital Imaging and Communications in Medicine) format.

Inclusion criteria were: fracture severity of at least B-Fracture according to AO (Arbeitsgemeinschaft Osteosynthese) Classification, no previous proximal tibial plateau fractures and complete preoperative image documentation that consisted of digital radiographic anteroposterior and lateral view of the knee as well as CT scans with maximum 1 mm axial slice thickness and multiplanar reconstructions in sagittal and coronal planes were mandatory.

Three Videos were generated from DICOM pictures (axial, sagital and coronal sequences of the CT scan) using Horos Software (Horos Viewer for Mac, v. 3.3.6, Horos Project, USA). Each video consisted of 40 images and were implemented in the online survey tool as scrollable frames. Thus, a User Interface (UI) was created similar to the in-house PACS System.

**Fig. 1:**
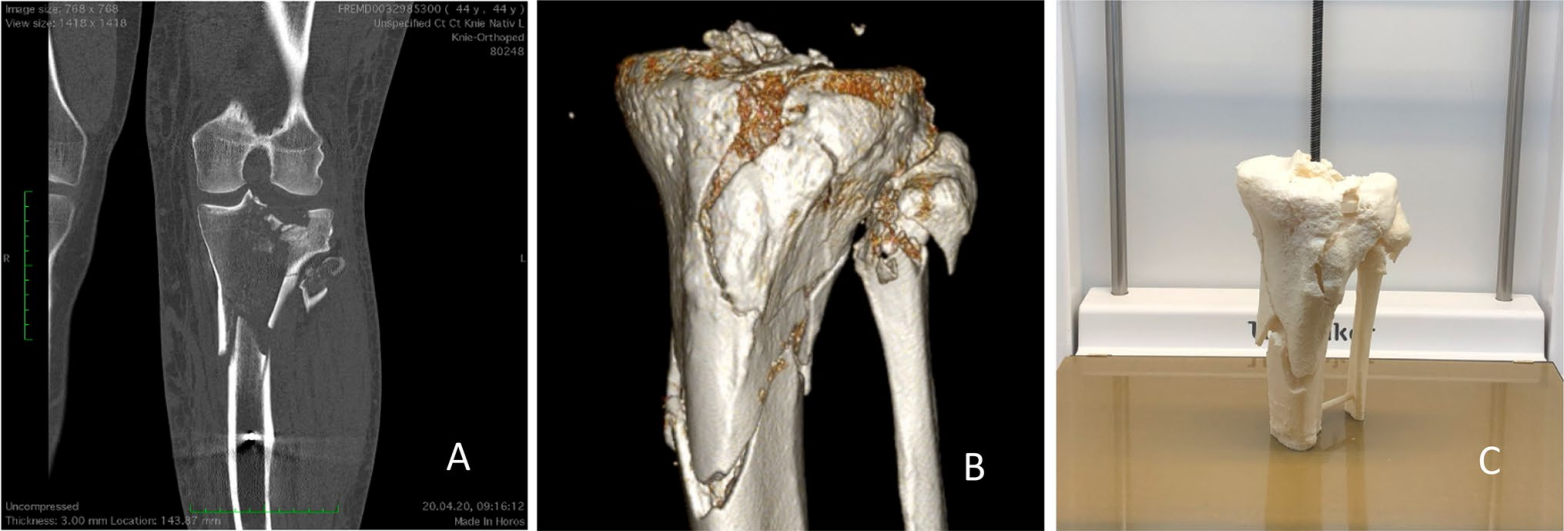
Tibial plateau fracture shown in CT (**A**), 3D reconstruction (**B**) and 3D-printed fracture model (**C**) imaging modalities

Using the same software bundle, two scrollable (transversal and longitudinal) 3D volumetric reconstructions of the proximal tibia and fibula with the subtraction of the femur and patella were generated consisting of 40 pictures and implemented in an online survey tool (Fig. 1).

### 3D print

CT scans of these fractures were saved as complete DICOM series and processed using Materialise’s Interactive Medical Image Control System (Mimics Innovation Suite v24; Materialise, Leuven, Belgium). Three-dimensional reconstructions were created using a threshold-based semi-automatic segmentation method with a threshold value of 226 and higher to separate soft tissue and to isolate bony structures. The femur and patella were digitally removed to enhance intra- articular fracture visualization. Due to impactation and fragmentation especially in complex, highly comminuted fracture types, some of the fragments had to be processed manually.

**Fig. 2:**
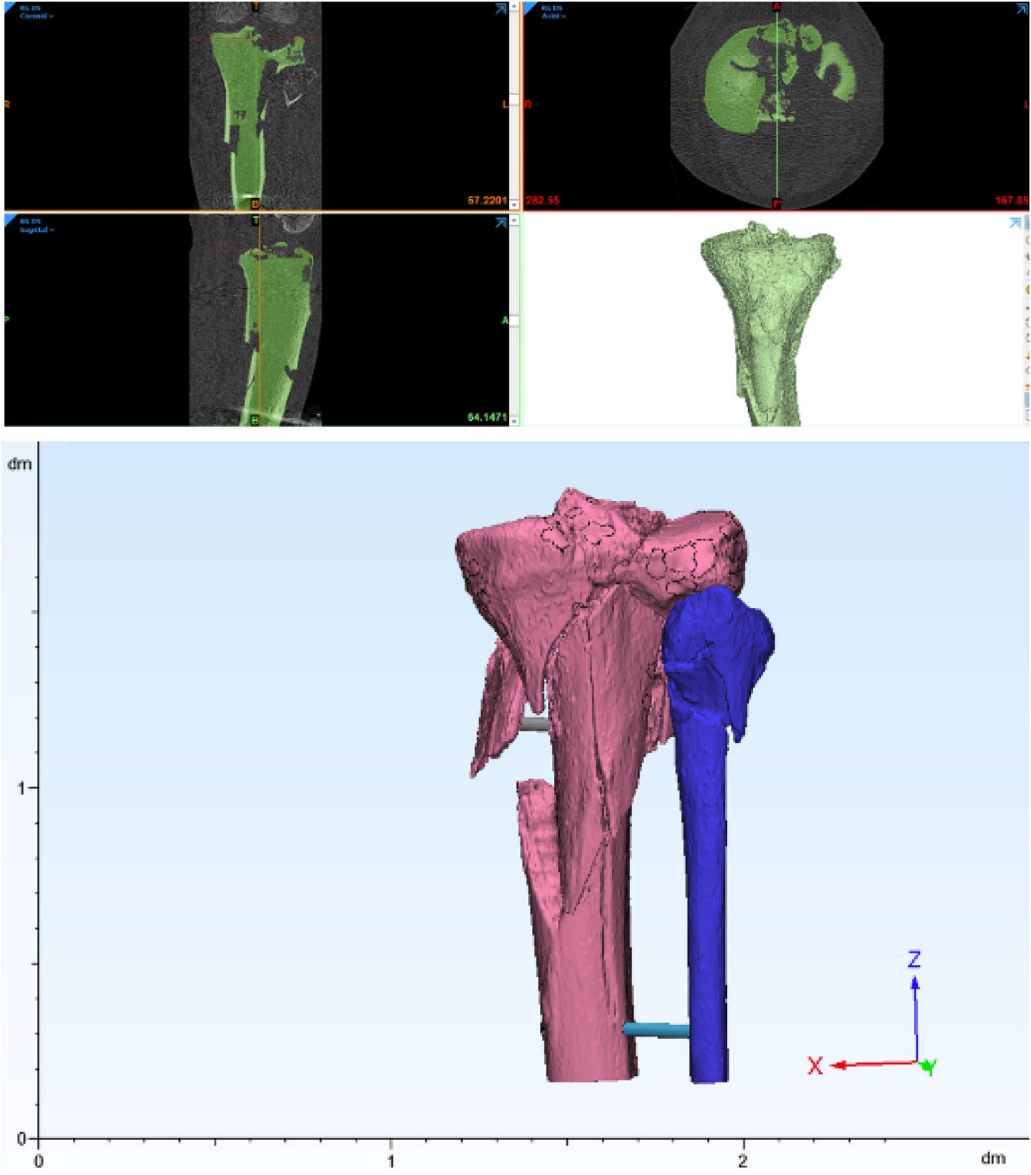
DICOM series processed using materialise’s interactive medical image control system (Mimics Innovation Suite v24; Materialise, Leuven, Belgium)

Segmented parts were exported to Materialise 3-Matic (Materialise 3-Matic Medical v16; Materialise, Leuven, Belgium) and post processed with global surface treatment and stabilization tubes for large fracture elements. A quick label was added and 3D reconstructions were exported as standard tessellation language (STL) (Fig. 2).

3D Printing was performed using an Ultimaker S5 Dual-Head Fused Deposition Modeling (FDM) Printer. This printer is a high-end FDM printer with a large build volume and offers the possibility to print simultaneously with two different print materials. Polylactic acid (PLA) was used for printing the fracture model, water soluble Polyvinyl alcohol (PVA) as support material.

For slicing process Cura (Ultimaker Cura v4.10; Ultimaker, Utrecht, Netherlands) was utilized. Layer height was set to 0.1 mm to ensure high level of detail. The STL file was converted into G-code to prepare the file for 3D printing. The tibial plateau fractures were printed on a scale of 1:1. After printing, the models required postprocessing to remove the support structures and brim.

### Online survey

**Fig. 3:**
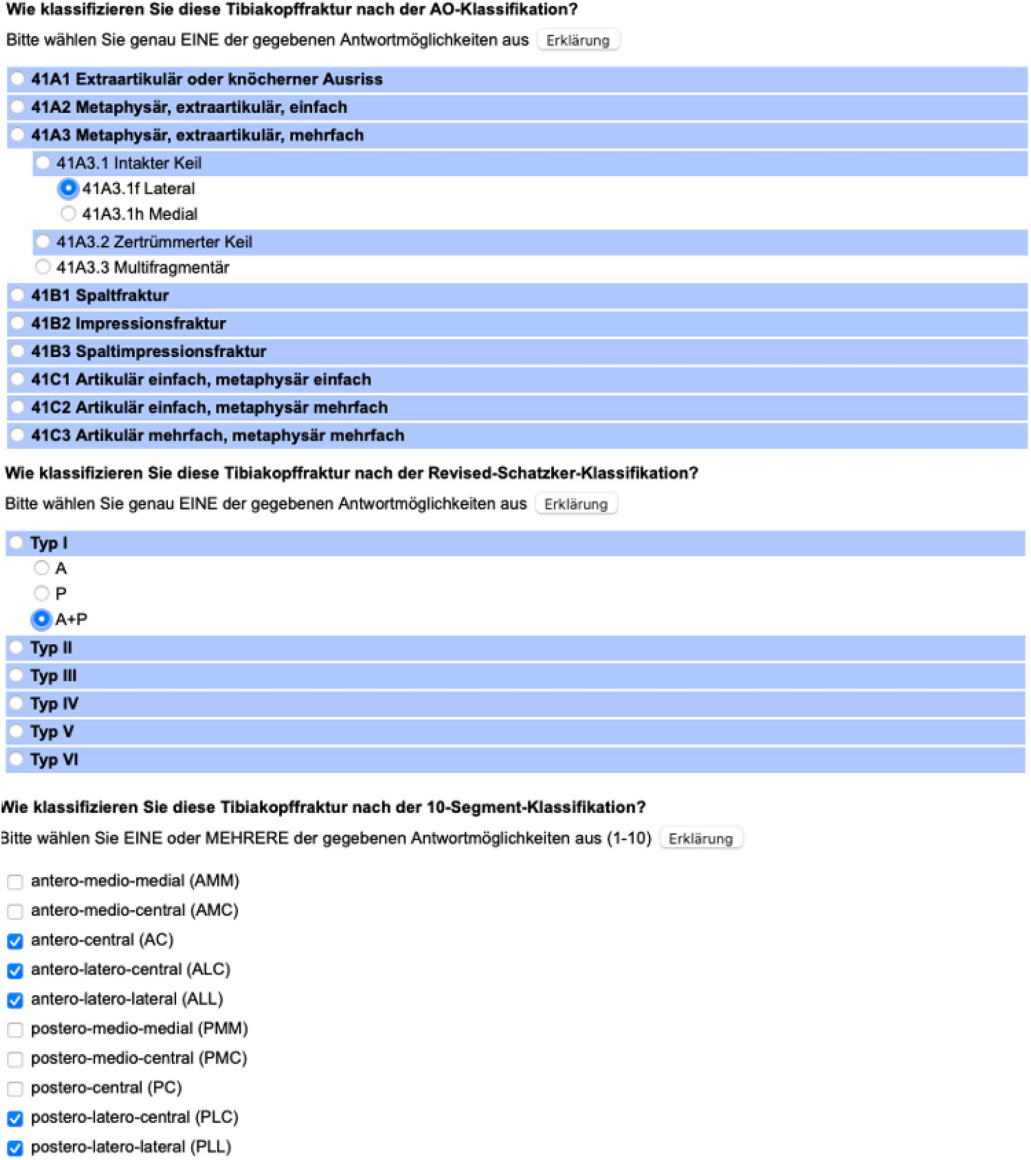
Classification survey within the online survey tool s2survey.net (SoSci Survey GmbH, Munich, Germany)

For each patient case a folder was generated using an online survey tool s2survey.net (SoSci Survey GmbH, Munich, Germany). This folder contained three scrollable interactive videos representing axial, sagittal and coronal plane of the patients CT sequences as well as two 3D reconstructions of the tibial fracture. Each page in one folder contained the fracture of one imaging modality and the questions regarding the different classifications and the subjective certainty (Fig. 3). These Folders were implemented at a web page at the online survey tool.

Twenty-two observers could be acquired and received an anonymous username and password to enter the online survey tool. All observers were provided with detailed instructions manual and were blinded to the cases. Due to different experiences of the observers with regard to clinical implementation of common tibial plateau fracture classification systems, information sheets with a brief classification overview were handed out to all the observers. The online survey contained a teaching of the systems at the beginning.

**Fig. 4:**
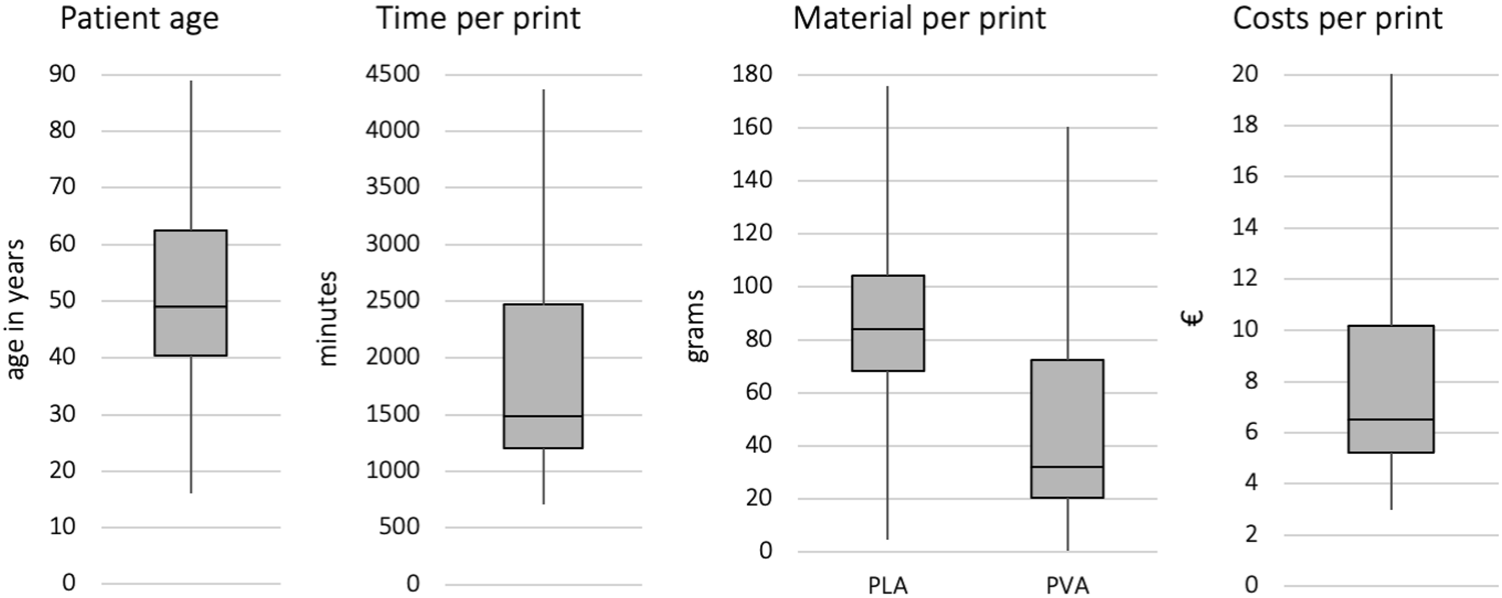
Descriptives of patient collective and print parameters

The following classification systems were included in the survey:

- Updated AO/OTA classification system [18]
- 10-Segment classification System [12, 19]
- Revisited Schatzker classification System [10]

The online questionnaire was designed to be completed in three different steps.

1. The observers were provided with plain transversal, sagittal and coronary CT slides and then asked to classify the fracture patterns
2. Two different 3D reconstructions, one rotatable by the y-axis and one by the x-axis, were used for classification.
3. The observers were asked to examine the matching 3D-printed specimen and completed the classification one final time. Next, they had to evaluate a potential benefit to classify a tibial plateau fracture through the 3D-printed specimen.

During each classification step, the observers were additionally asked how confident they were about their decision using a five-step Likert scale. After the completion of each step the observers were unable to reach the previous webpage to alter previous results. Each question was displayed as a multiple-choice answer with expanding drop-outs.

Once the survey was completed, the account of each observer was locked to prevent subsequent cross-checking. After at least four weeks, ten randomly selected observers–equally distributed over the different levels of professional experience–received a new personalized and anonymized password-locked invitation to re-run the entire survey a second time with the case order newly randomized. To ensure valid intraobserver reliability testing, the number of retested observers was determined based on previously published relevant studies on fracture classification [20–23]. Institutional review board ethical approval was obtained before study initiation.

### Statistics

All answers were stored in an online database and then exported to Microsoft Excel (Microsoft Office 365, Microsoft Corporation, Redmond, USA). Descriptive statistics were used to analyze parameters of the patient collective and printing process. Kappa coefficient was calculated and interpreted according to the criteria proposed by Landis and Koch (Table 1) to analyze the inter- and intraobserver reliability of the AO/OTA, 10-Segment and Revisited Schatzker classification at every stage of the three steps [24–26]. Further percentage match (“PM”) was used to provide an additional measurement for the interobserver reliability [27]. To quantify the subjective certainty of the decisions made by the rater, five-step Likert-Scales were used and a univariant one-way analysis of variance (ANOVA) to determine whether there were significant differences between the groups (CT vs. 3DCT vs. 3D-print).

**Table 1:** Landis and Koch grading of reliability based on kappa coefficient subjective certainty regarding the diagnostical decisions made by the rater

*Κ* coefficient	Reliability grading
< 0.00	Poor
0.01–0.20	Slight
0.21–0.40	Fair
0.41–0.60	Moderate
0.61–0.80	Substantial
> 0.80	Excellent

## Results

Twenty-two patients (seven females and fifteen males, mean age 49 ± 19 years) with tibial plateau fractures were enrolled and analyzed by 22 raters with different levels of experience. Eleven unicondylar partial articular- and eleven bicondylar complete articular tibial plateau fractures were printed using an Ultimaker S5 FDM 3D printer with a mean printing time of 32:11 h (± 15:46 h). The mean weight of the models was 137.6 g (± 67.1 g) with 68.5% being the main PLA material and 31.5% being the PVA Natural support material (Fig. 4).

### AO classification

The overall reliability of the AO classification showed a fair agreement for all modalities (CT 0.32; 3DCT 0.32; 3D-print 0.34) according to the criteria proposed by Landis and Koch [22]. The overall percentage match increased from 41% (CT and 3DCT) to 43% (3D-print), with the senior surgeons showing the biggest improvement, raising their PM 7% from 41 to 48% (Table 2). Both, Medical students’ and surgical residents’ kappa values increased by 0.03, whereas junior and senior surgeons improved their respective kappa values by 0.08 from 0.26 to 0.34 and from 0.30 to 0.38. The intraobserver agreement kappa values for the AO classification showed a moderate agreement with the overall value being 0.44. The 3D-print specific value was 0.47 – scoring the highest value out of all the different imaging modalities (Table 5). Further the raters changed their classification in 23% of the cases after evaluating the fracture with the 3D-print (Table 6).

**Table 2:** Interobserver agreements for the AO classification system

AO classification	CT		3DCT		3D	
	PM	*Κ*	PM	*Κ*	PM	*Κ*
Overall	41%	0.32	41%	0.32	43%	0.34
Medical students	48%	0.38	49%	0.39	50%	0.41
Surgical residents	41%	0.32	39%	0.30	43%	0.35
Junior surgeons	38%	0.26	42%	0.32	44%	0.34
Senior surgeons	41%	0.30	41%	0.31	48%	0.38

### 10-Segment classification

The analysis of the kappa coefficient for the 10-Segment classification showed, overall, a slight agreement, improving from 0.11 (CT and 3DCT) to 0.18 (3D-print). The medical students showed the biggest improvement of 0.10, increasing their value from 0.09 (CT) to 0.19 (3D-print). The percentage match improved 8% from 71 to 79% overall agreement, when viewed by CT, 3DCT and 3D-print. For medical students the PM improved 10% from 71% (CT) to 81% (3D-print) and for surgical residents it improved from 71% (CT) to 78% (3D-print). The junior and senior surgeons were able to improve their PM values by 2% and 7% (Table 3). The intraobserver agreement values showed a fair agreement for all the modalities with the overall kappa value being 0.27 (Table 5). For the 10-Segment classification the raters changed their classification in 55% of the cases after 3D-print evaluation.

**Table 3:** Interobserver agreements for the 10-segment classification system

10-Segment classification	CT		3DCT		3D	
	PM	*Κ*	PM	*Κ*	PM	*Κ*
Overall	71%	0.11	76%	0.11	79%	0.18
Medical students	71%	0.09	76%	0.11	81%	0.19
Surgical residents	71%	0.10	75%	0.10	78%	0.19
Junior surgeons	74%	0.07	78%	0.05	76%	0.11
Senior surgeons	74%	0.14	79%	0.15	81%	0.18

### Revisited Schatzker classification

The Revisited Schatzker classification showed, overall, a fair agreement in the kappa coefficient analysis, with the CT specific kappa value being 0.24, the 3DCT value being 0.28 and 3D-print value being 0.31. The percentage match analysis for the Revisited Schatzker classification resulted in an overall agreement of 32% (CT), 33% (3DCT) and 35% (3D-print). The junior and senior surgeons were not able to improve their PM. Their values decreased by 1% and 3% from 33% (CT) to 32% (3D-print), respectively from 27% (CT) to 24% (3D-print) (Table 4). The kappa evaluation showed the junior and senior surgeons again decreasing their interobserver agreement kappa values by 0.02 and 0.04, with the senior surgeons even performing a kappa class switch from a fair to a slight agreement according to the criteria by Landis and Koch [22]. Their value decreased from 0.22 (CT) to 0.18 (3D-print). The medical students and surgical residents on the other hand were able to increase their values by 0.05 and 0.06, respectively. The intraobserver values showed a moderate agreement with the 3D-print specific value scoring the highest agreement value (0.47) of all the imaging modalities (CT 0.38 and 3DCT 0.41) (Table 5). With the Revisited Schatzker classification the raters changed their classification in 31% of the cases after fracture evaluation with the 3D-printed specimen (Table 6).

**Table 4:** Interobserver agreements for the revisited Schatzker classification system

Revisted Schatzker classification	CT		3DCT		3D	
	PM	*Κ*	PM	*Κ*	PM	*Κ*
Overall	32%	0.24	33%	0.28	35%	0.31
Medical students	35%	0.30	38%	0.33	40%	0.35
Surgical residents	32%	0.25	33%	0.27	35%	0.31
Junior surgeons	33%	0.27	33%	0.27	32%	0.25
Senior surgeons	27%	0.22	28%	0.23	24%	0.18

**Table 5:** Intraobserver agreements for the AO, 10-segment and revisited Schatzker classification system

Intraobserver	CT	3DCT	3D	Overall
	*Κ*	*Κ*	*Κ*	*Κ*
AO classification	0.42	0.42	0.47	0.44
10-Segment classification	0.25	0.27	0.26	0.27
Revisited Schatzker classification	0.38	0.41	0.47	0.42

**Table 6:** Percentage change in classification after 3D-print evaluation

Change in classification	%
AO classification	23%
10-Segment classification	55%
Revisited Schatzker classification	31%
Total	36%

### Subjective certainty

The analysis of the five-step Likert-scales showed that the subjective perceived certainty of the rater regarding their classification significantly improved for all three classification systems. Categories of self-confident diagnostic choice were selected 20%, 18% and 16% more often for the AO classification (46% > 47% > 66%; *p* < 0.05), 10-Segment classification (41% > 43% > 59%; *p* < 0.05) and Revisited Schatzker classification (47% > 48% > 63%; *p* < 0.05) while classifying the fractures with the 3D prints (Table 7).

**Table 7:** Subjective certainty regarding the diagnostical decisions made by the rater

Category: safe and very safe	CT	3DCT	3D	*p* value
AO classification	46%	47%	66%	< 0.05
10-Segment classification	47%	48%	63%	< 0.05
Revisited Schatzker classification	41%	43%	59%	< 0.05

### Informative benefit

In 76% of the cases the raters stated that they gained additional information using 3D print fracture evaluation.

## Discussion

In contrast to previous studies on two-dimensional based tibial plateau fracture classification systems using conventional imaging techniques, this study was the first to investigate the reliability of modern CT-based axial fracture classification systems accompanied by the support of 3D-printed fracture models. Not only the reliabilities of the individual classification systems among each other were compared but also the application by surgeons with different levels of professional experience.

Overall, the AO classification and the Revisited Schatzker classification showed a fair interobserver reliability regarding the kappa values while the 10-Segment classification showed a slight interobserver reliability. However, the 10-Segment classification proclaimed a significantly higher reliability regarding the percentage match (PM) evaluation than the other classification systems. Furthermore, three-dimensional printouts of tibial plateau fractures improved inter- and intraobserver agreements and significantly increased the raters’ confidence with their decision. In addition, in 76% of the cases, the raters obtained an informative benefit for the surgical management of the patient using a 3D-printed fracture model.

For proper preoperative planning, a correct fracture classification is of substantial importance. Fracture specific classification systems that provide high inter- and intraobserver agreement values may be used [28, 29]. In 2018 Millar et al. concluded in their extensive review of classificational systems of the tibial plateau that the use of CT scans increases the interobserver agreement of those systems [13, 26]. However, the majority of those analyzed systems were developed using two-dimensional roentgenographic imaging [7, 8]. By classifying fractures with three-dimensional imaging modalities this may lead to detection of fracture specifics not identifiable by planar radiographs especially in the posterior tibial plateau column. Waldrop et al. were able to show that fractures of the posterior aspects of the tibial plateau are not reliably and sufficiently displayed in two-dimensional planar imaging and therefore can lead to postoperative knee instability and malreductions [30]. In contrast, the modern systems provide a sufficient basis for proper identification and representation of those types of fracture characteristics [10, 12].

The novel CT-based axial classification systems are significantly more complex than the two-dimensional-based systems with more choices for fracture description. Especially for complex fracture patterns, it has been shown that the disagreement among raters increases [13, 31–33]. Thus, the interrater reliabilities of the individual classification systems with mostly “slight” or “fair” kappa values show a comparatively slightly lower reliability.

In addition to the increased complexity, by including a total of twenty-two raters this survey was poised to score rather lower kappa values as the chance of a higher agreement decreases with an enlargement of the number of raters evaluating the fracture. But, with so many raters included a high level of redundancy for the measured outcomes was ensured. Different outcomes of comparable studies may be due to a different number of cases, raters, varying levels of pretest teaching and due to quality improvements of imaging methods that may increase the chance of agreement [13]. To increase the comparability between the classification systems and the CT/3D printing group, an additional reliability analysis was performed using the percentage match agreement.

Several authors have suggested that different clinical experience levels may affect the interobserver reliability [32, 34, 35]. However, this study has shown that different levels of experience could influence interobserver reliability concerning all three classification systems.

For the AO classification an increase regarding the inter- and intraobserver agreement was found across all levels of experience with the senior surgeons showing the highest improvement. The classification itself provides a detailed pictogram of every specification. That may have helped the raters in their decision-making process [18]. Another important factor is the level of detail that the raters were asked to answer, as it was a requirement to pick among the subgroups with the use of qualifiers.

The 10-Segment classificatons inter- and intraobserver agreement evaluation using the kappa coefficient has some weaknesses since the classification system has a multitude of different items. An agreement in nine out of ten selected segments will statistically be evaluated as disagreement leading to low kappa values. Thus the 10-Segment classification showed the highest agreement according to the percentage match analysis while scoring the lowest kappa values. This discrepancy between the comparatively low kappa and high PM values suggests that the observers showed only minimal differences when classifying individual segments of the 10S classification during fracture morphology evaluation. With regard to an accurate and reliable preoperative diagnosis of severe tibial plateau fractures, the clinical benefit of the 10-segment classification, based on the consistently high percentage match values across all experience levels of the 22 raters, seems to be significantly higher in comparison to the AO and Revisited Schatzker classification system. This matches the high intra-and interobserver reliability observed in other studies [36].

Prior studies mostly included the original Schatzker classification in their analysis of the overall agreement in classification [8]. In 2018 Kfuri et al. published an updated version of the Schatzker classification–introducing a virtual equator, dividing the tibial plateau not only in medial and lateral columns but also in anterior and posterior aspects [10]. The analysis of the revisited Schatzker classification showed a consistent increase of agreement in the two less experienced rater groups. In contrast, the two more experienced rater subgroups had lower agreement values with improving intraobserver agreements. After evaluating the prints, the raters changed their decisions in one third of the cases and improved their subjective perceived certainty regarding their decisions. All these improvements may be due to a haptic fracture understanding.

Even though the Revisited Schatzker and 10-Segment classification allow for very precise locating of fracture parts they fail to adequately display fracture morphology and their displacement (split or depression) [13]. In contrast, the AO classification takes those aspects better into account while providing a less accurate location determination [33].

While an accurate and reliable fracture classification and hereby adequate representation of highly variably and complex fracture pattern is relevant, one of the main goals should be agreement on a precise preoperative planning of the patient specific surgical treatment [13]. The evaluation in multiple plains has proven to help preoperative planning and to find the ideal surgical approach. Further, it helps to improve fracture reduction. This is of substantial importance for the patient's outcome [13, 28, 37]. In comparison to the two-dimensional based classification systems the modern systems emphasize such concepts [10, 14]. The main advantage of the axial fracture assessment is the evaluation of involvement of anterior or posterior fracture aspects in combination with the lateral and medial aspects. Hereby, the modern systems provide a better basis for adequate preoperative planning [10, 12, 13, 38].

### 3D print

The majority of the existing studies regarding the inter- and intraobserver agreement of classification systems of the tibial plateau, mostly only investigated conventional imaging techniques such as plain radiographs, computed tomography scans or 3D volumetric reconstructions. In contrast, this study introduces the novel 3D printing technique for preoperative diagnostics which has been validated as a precise complement for correct imaging of the human anatomy [39].

Several studies have demonstrated the advantage of a 3D-printed fracture model over CT imaging alone for the diagnosis of complex fractures [40, 41]. This study showed, that the implementation of point of care 3D-printing may provide additional value when compared to the sole use of conventional imaging modalities. Especially in clinically less experienced raters, the 3D-print group showed higher interobserver reliability than CT and 3DCT alone. Brouwers et al. demonstrated similar results in their study dealing with acetabular fractures [42]. But it remains unclear whether the use of 3D printing also has a positive impact on preoperative planning in young inexperienced surgeons.

In the process of the production of a 3D-printed specimen the time-factor needs to be considered. The average time of production for one of the models took between one and one and a half days. At least in the practice of the authors, tibial plateau fractures are not routinely treated immediately but around five to eight days after trauma [43, 44]. When introducing 3D printing technology in daily clinical practice this meantime may be used for production. However, default routines are needed to be implemented to ensure a timely start of production in every case. A crucial detail is, that personnel is required with specialized training, to ensure a consistent quality in the data preparation and printing process.

Another important aspect in the findings of this study, is that especially young and inexperienced surgeons as well as medical students particularly benefited from the 3D-printed models. This group showed the greatest learning effect with the 3D models during repeated study execution. Li et al. could show in a systematic review that also in the field of neurosurgical applications a significant learning effect on the part of medical students could be seen by the supporting application of 3D-printed models [45].

A number of limitations need mentioning: To ensure radiation protection and for ethical reasons, patients referred from smaller hospitals did not get a second CT scan at the investigating site for scientific purposes. The cases and their external CT datasets were included in this study if they met the technical requirements for 3D printing. In theory, this may have led to quality differences among the prints. Then, Wainwright et al. proposed the existence of a fatigue factor after evaluating a multitude of radiographic images which could lead to decreasing detail perception after some time [46]. To overcome this issue, the raters were offered the possibility to suspend the survey at any given page and to resume later. In the end, the existence of a fatigue factor potentially biasing the data presented cannot be excluded with absolute certainty.

## Conclusion

Overall, the AO classification and the Revisited Schatzker classification show a fair kappa reliability according to the criteria proposed by Landis and Koch [22], while the 10-Segment classification shows a slight reliability. However, the percentage match evaluation shows the strongest data for the 10-Segment classification (79%), while the AO classification and Revisited Schatzker classification scored an overall value of 43% and 35%. Furthermore, this investigation shows a substantial benefit for the diagnostics of tibial plateau fractures using 3D printing technology. The resulting benefits in the preoperative planning process may have the potential to improve postoperative outcomes, too. The time-consuming production of a single print may currently challenge seamless preoperative processes and will require thorough planning until further technical advances will help accelerate printing speeds in the future.

Overall, the 10-segment classification seems to provide a good introduction to the broad field of fracture surgery at the knee joint, especially for inexperienced surgeons. An additional preoperative 3D print allows a more profound understanding of the fracture morphology and ultimately the necessary surgical approach.
